# Alumni Associations

**Published:** 1881-03

**Authors:** Walter S. Harban

**Affiliations:** Recording Secretary


Alumni Associations.—Meetings were held soon after the commencement exercises were concluded, in both institutions, and the graduates, old and young, both of Doctors and Dentists, seem to have had a "good time" at their respective festivals. We have received the following official report :

The Sixth Annual Meeting of the Alumni Association of the Baltimore College of Dental Surgery, was held in the lecture room of this College, March 2nd, 1881.

The regular order of business was dispensed with to elect officers for the ensuing year; which resulted in the election of Dr. F. J. S. Gorgas, President; Dr. L. M. Cowardin, First Vice-President; Dr. Basil M. Wilkerson, Second Vice-President; Dr. T. S. Latimer, Treasurer; Dr. T. E. Craddock, Corresponding Secretary; Dr. Walter S. Harban, Recording Secretary, and Dr. Holly Smith the Orator. The meeting adjourned without transacting any other business except the adoption of a motion providing for the Annual Alumni Banquet. WALTER S. HARBAN, *Recording Secretary.*

